# 872. Characterizing Plasma Microbial Cell-Free DNA Sequencing Detections of Public Health Important Pathogens

**DOI:** 10.1093/ofid/ofad500.917

**Published:** 2023-11-27

**Authors:** Sarah Y Park, Eliza J Chang, Constance Lau, David Berman, Frederick S Nolte

**Affiliations:** Karius, Redwood City, California; Karius, Redwood City, California; Karius, Inc., Redwood City, California; Karius, Redwood City, California; Karius, Redwood City, California

## Abstract

**Background:**

Timely pathogen identification is critical for meaningful disease surveillance to facilitate effective public health responses. However, public health important pathogens are often diagnostically challenging (e.g., indeterminate serological results, multiple samples separated by long intervals, lengthy turnaround times) and at odds with public health needs. We describe detections of public health important pathogens by the Karius Test®, a validated plasma microbial cell-free DNA (mcfDNA) sequencing test, providing real-time pathogen detection.

**Methods:**

We identified nationally notifiable infectious diseases (NNID)-associated and selected state reportable pathogens detected and quantified as DNA molecules/μl (MPM) in US patients’ plasma samples submitted to the Karius CLIA certified/CAP accredited laboratory for mcfDNA sequencing between 4/1/18–4/30/22. We evaluated and characterized these detections based on result and test requisition form data. We reviewed gender and immunocompromised status of a subset of patients linked deterministically to the Premier Healthcare Database (PHD), a US hospital-based, all-payer database.

**Results:**

Plasma mcfDNA sequencing yielded 520 detections of 62 microbes associated with 27 NNIDs and 449 detections of an additional 12 microbes, including public health important parasites, amoebae, endemic mycoses, and selected bacteria, across 27 US states. MPM and patient age varied widely across microbes from 9–300+ million and 0–89 years, respectively (Table 1). For the 167 (17%) PHD-linked detections, 71% were associated with male and 72% with immunocompromised patients.

**Figure:**
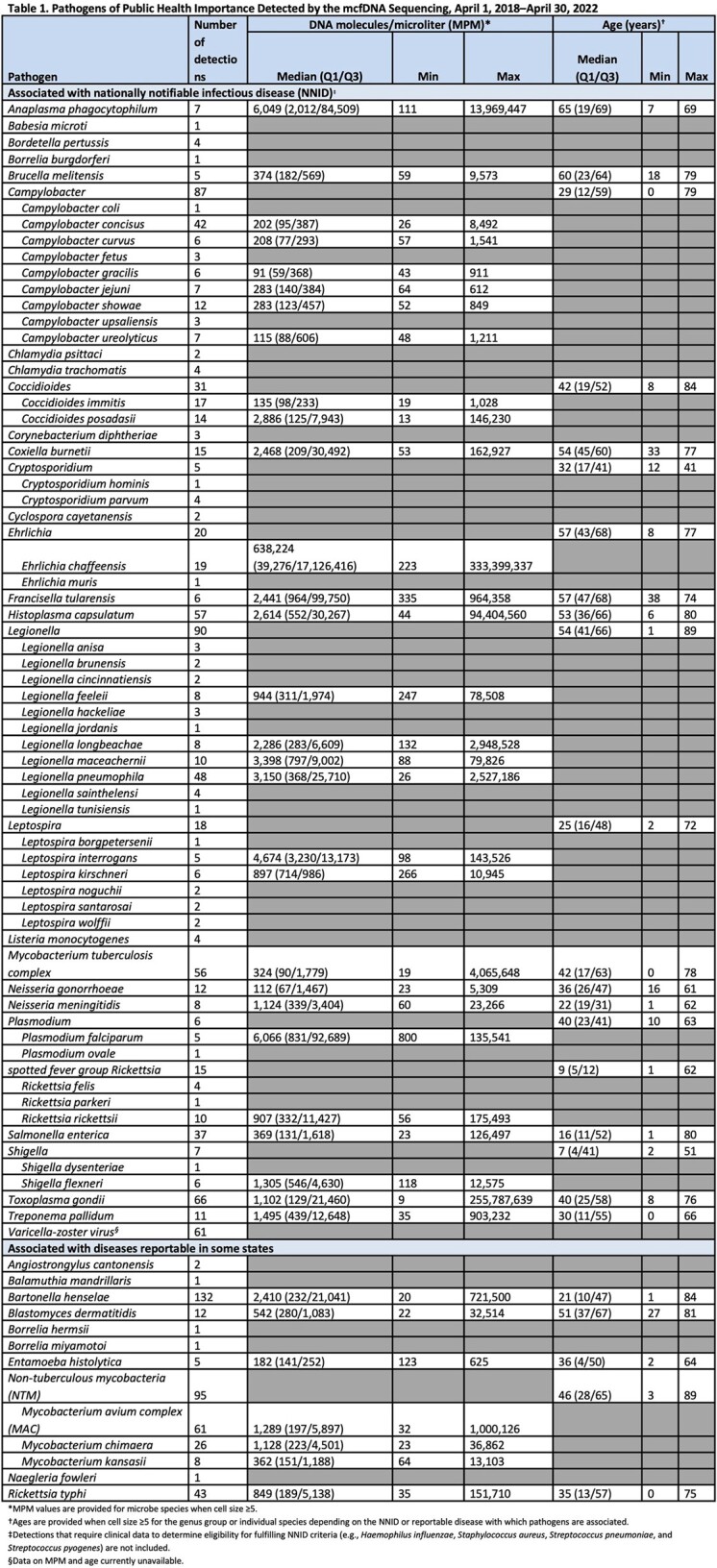

**Conclusion:**

Plasma mcfDNA sequencing detected many different public health important pathogens across many states, especially among immunocompromised patients. These patients pose a heightened public health concern given their increased risk for more severe disease and potential for longer infectious periods and greater likelihood of atypical or nonspecific presentations, leading to greater risk of unrecognized disease spread. Plasma mcfDNA sequencing, an unbiased test, may contribute to rapidly identifying disease outbreaks and spread, and facilitate timely interventions to improve public health.

**Disclosures:**

**Constance Lau, MPH**, Karius, Inc.: Stocks/Bonds **David Berman, DO**, Precision Health Solutions, St. Petersburg, Florida: Advisor/Consultant **Frederick S. Nolte, PhD**, Karius: Stocks/Bonds

